# Effectiveness of guideline dissemination and implementation strategies on health care professionals’ behaviour and patient outcomes in the cancer care context: a systematic review

**DOI:** 10.1186/s13012-020-0971-6

**Published:** 2020-06-03

**Authors:** Jennifer R. Tomasone, Kaitlyn D. Kauffeldt, Rushil Chaudhary, Melissa C. Brouwers

**Affiliations:** 1 School of Kinesiology & Health Studies, Queen’s University, 28 Division Street, Kingston, Ontario Canada; 2 Department of Medicine, University of Toronto, 27 King’s College Circle, Toronto, Ontario Canada; 3 School of Epidemiology and Public Health, University of Ottawa, 600 Peter Morand Crescent, Ottawa, Ontario Canada

**Keywords:** Clinical practice guidelines, Cancer care, Dissemination, Implementation, Medical health care professionals, Allied health care professionals

## Abstract

**Background:**

Health care professionals (HCPs) use clinical practice guidelines (CPGs) to make evidence-informed decisions regarding patient care. Although a large number of cancer-related CPGs exist, it is unknown which CPG dissemination and implementation strategies are effective for improving HCP behaviour and patient outcomes in a cancer care context. This review aimed to determine the effectiveness of CPG dissemination and/or implementation strategies among HCPs in a cancer care context.

**Methods:**

A comprehensive search of five electronic databases was conducted. Studies were limited to the dissemination and/or implementation of a CPG targeting both medical and/or allied HCPs in cancer care. Two reviewers independently coded strategies using the Mazza taxonomy, extracted study findings, and assessed study quality.

**Results:**

The search strategy identified 33 studies targeting medical and/or allied HCPs. Across the 33 studies, 23 of a possible 49 strategies in the Mazza taxonomy were used, with a mean number of 3.25 (SD = 1.45) strategies per intervention. The number of strategies used per intervention was not associated with positive outcomes. Educational strategies (*n* = 24), feedback on guideline compliance (*n* = 11), and providing reminders (*n* = 10) were the most utilized strategies. When used independently, providing reminders and feedback on CPG compliance corresponded with positive significant changes in outcomes. Further, when used as part of multi-strategy interventions, group education and organizational strategies (e.g. creation of an implementation team) corresponded with positive significant changes in outcomes.

**Conclusions:**

Future CPG dissemination and implementation interventions for cancer care HCPs may benefit from utilizing the identified strategies. Research in this area should aim for better alignment between study objectives, intervention design, and evaluation measures, and should seek to incorporate theory in intervention design, so that behavioural antecedents are considered and measured; doing so would enhance the field’s understanding of the causal mechanisms by which interventions lead, or do not lead, to changes in outcomes at all levels.

Contributions to the literature

- This paper is the first comprehensive review of the effectiveness of guideline dissemination and implementation strategies in the cancer care context and builds upon previous reviews of this topic in general contexts.
- The findings of this review have the potential to inform researchers and practitioners who are interested in strategies that enhance guideline dissemination and implementation, and thus the quality and safety of cancer health care, among both medical and allied health care professionals.

## Background

Clinical practice guidelines (CPGs)—‘statements that include recommendations intended to optimize patient care […] informed by a systematic review of evidence and an assessment of the benefits and harms of alternative care options’—are commonly regarded as tools for quality improvement in health care [1]. CPG recommendations are based on the syntheses of scientific evidence (i.e. trials of clinical effectiveness; e.g. a given course of chemotherapy is effective at reducing a given tumour) and have been critically appraised by experts in the respective field [1]. Accordingly, in line with Proctor et al.’s [5] conceptual model of implementation research, CPGs represent a tool for evidence-based practice; when adopted and used by health care professionals (HCPs), CPGs have the potential to improve health care service delivery (i.e. HCP behaviour) and, subsequently, patient outcomes [2]. However, the development of CPGs alone does not guarantee a change in HCP behaviour or patient outcomes [3]; dissemination strategies (i.e. targeted distribution of CPG information and materials) and implementation strategies (i.e. techniques to enhance CPG adoption, use and sustainability) [2] are required, and must be effective, for CPGs to impact health care delivery and outcomes [4, 5].

Two reviews have examined the effectiveness of CPG dissemination and implementation strategies for medical and allied HCPs^1^ [6, 7]. Both reviews reported that passive educational strategies and identifying barriers to CPG implementation were frequently paired with modest improvements in HCP behaviour when used alongside other strategies in a given intervention.^2^ However, these reviews also concluded that sufficient evidence does not exist to support a ‘universal’ CPG dissemination and/or implementation strategy for medical or allied HCPs [6, 7], as the effects of such strategies vary greatly across trials. While findings from Grimshaw et al. [7] and Hakkennes and Dodd [6] offer a foundation for understanding CPG dissemination and/or implementation strategies among HCPs, they are based on papers that were published up to 1998 and 2006, respectively, and neither are specific to the cancer care context. More recently, attempts have been made to synthesize terminology [8, 9] and evidence [10] for dissemination and implementation strategies in order to manage the breadth and volume of published literature in this emerging field. Incomplete reporting of intervention details and a lack of consideration of the specific context in which care is provided continue to challenge these efforts [11].

Current evidence suggests that medical and allied HCP adherence to CPGs in cancer care is sub-optimal [12–14]. The cancer care context poses a unique challenge to medical and allied HCPs in that effective patient care requires expertise from multiple medical (e.g. nurses, oncologists) and allied (e.g. social worker, physiotherapists) HCPs operating as a coordinated health care team [15]. Cancer services are often provided across different settings (e.g. community, clinical, inpatient) and require individuals to transition between care providers and care settings over the course of their disease trajectory (e.g. screening, diagnosis, treatment, follow-up/surveillance) [16]. Moreover, cancer research is constantly evolving, and CPGs require routine and frequent updating to ensure best-practice recommendations remain current and valid [17]. Further, with the emergence of new technologies (e.g. electronic medical records) and treatments (e.g. chemotherapy regimens), patient care within the cancer context is constantly advancing [18]. As a result, adopters of CPG recommendations (i.e. medical and allied HCPs) are required to continually be aware of developments in CPGs in order to deliver safe, effective, and evidence-based patient care. Given the unique challenges posed to medical and allied HCPs in cancer care, it is critical that CPGs are disseminated and implemented using strategies that are effective in this context. Thus, to inform future intervention design, an updated exploration of effective CPG dissemination and implementation strategies within the cancer context is warranted. Accordingly, the aim of this review was to determine the effectiveness of CPG dissemination and implementation strategies among HCPs in the cancer care context.

## Methods

This review was conducted according to the Preferred Reporting Items for Systematic Reviews and Meta-Analyses (PRISMA) statement. The protocol was registered with the PROSPERO database (CRD 42015019331) and previously published [19].

### Literature search strategy and selection

Five electronic databases (MEDLINE, EMBASE, PsychINFO, CINAHL, and the Cochrane Controlled Trials Register) were systematically searched using a search strategy developed from previous systematic reviews examining CPG dissemination and/or implementation strategies in medical and/or allied health care contexts [6, 7], and the Cochrane Effective Practice and Organization of Care (EPOC) Group’s strategy [8]. The search combined search terms relevant to CPG dissemination and implementation, medical and allied HCPs, outcomes, trial design, and cancer care. The search strategy was peer-reviewed by a health sciences librarian external to the research team, with expertise in systematic review searching. The full search strategy, inclusive of all search terms, has been previously published [19] and is available in Additional file 1.

Search results were limited to studies written in the English language, with human subjects, and published between 1998 (date of the last systematic review examining effectiveness of CPG dissemination and implementation strategies among medical HCPs [7];) and March 2018. Search items were screened for eligibility, and the reference lists of systematic reviews and/or meta-analyses identified through the search were hand-searched to confirm literature saturation.

#### Inclusion/exclusion criteria

Inclusion criteria for this review were that studies had to (a) be published in a peer-reviewed journal; (b) use an experimental (randomized controlled trial (RCT), controlled clinical trial), or quasi-experimental study design (interrupted time series, controlled before-and-after design); and (c) examine the dissemination and/or implementation of CPGs among medical and/or allied HCPs within the cancer care context. In line with similar reviews [6, 7], CPGs were broadly operationalized as clinical guidelines, practice guidelines, guidance, advice, recommendations, expert opinion, and consensus statements.

Exclusion criteria included (a) cross-sectional, cohort, case, and retrospective study designs; (b) unpublished data, abstracts, conference proceedings, and qualitative only studies; (c) studies exclusively targeting the dissemination and/or implementation of CPGs among patients, the general public, and hospital administrators; and (d) studies that were not designed to enhance the dissemination and/or implementation of a CPG (i.e. clinical interventions, such as those testing a new treatment modality that aim to establish clinical effectiveness).

#### Outcome(s) included

The outcome of interest was an objective or subjective (i.e. self-reported) measure of HCP behaviour (i.e. service delivery [5];) in relation to CPG recommendations, such as screening rates, prescription, or referrals. Antecedents to behaviour—such as knowledge, attitudes, or perception of barriers towards a CPG—were also considered as primary outcomes. Secondary outcomes (i.e. outcomes that stem from a change in service delivery/HCP behaviour) included patient outcomes such as survival, quality of life measures, test completion, and pain.

#### Screening process

Bibliographic records from each database were uploaded into EndNote X7 reference management software. Duplicates were removed through the comparison of citation details, such as author names, year of publication, article title, and journal. The titles and abstracts of potentially relevant studies were screened independently by two reviewers. The full text of studies passing the initial screening level was examined by two independent reviewers to verify eligibility. Reviewer consensus was required at both levels of screening for study inclusion. Any discrepancies were resolved through reviewer discussion and consultation with the first author when required.

### Data extraction

The first and third authors developed a data extraction form using the data collection checklist by Grimshaw et al. [7], with appropriate modifications made for the cancer care context. The following data was extracted from each eligible full-text study: (a) study design, (b) quality criteria, (c) characteristics of participating HCPs, (d) characteristics of participating patients, (e) intervention setting (e.g. outpatient or inpatient), (f) intervention characteristics (e.g. CPG characteristics, and dissemination and/or implementation strategies used), and (g) outcomes (e.g. HCP behaviour, patient outcomes). The data extraction form was piloted independently by two reviewers and revised prior to full extraction of included studies. All data were extracted by a single reviewer and verified by a second reviewer. Any notable discrepancies were resolved through discussion and consultation with the first author when required. The reviewers were not blinded to the purposes of this review.

Early in the extraction process, reviewers noted that (a) in addition to HCP-directed strategies, a number of studies included patient-directed (i.e. aiming to promote patients’ involvement in health care) or patient-mediated (i.e. aiming to enhance HCP behaviour through patient-provider interaction) dissemination and/or implementation strategies, which can influence HCP behaviour and/or patient outcomes [20], and (b) theory use was uncommon within the included studies, despite the importance of theory for the development and evaluation of complex health system interventions [21]. Thus, reviewers deviated from the original review protocol and noted whether (a) the intervention also included patient-directed and/or patient-mediated strategies, and (b) theory was used for intervention design and/or evaluation [19].

#### Mazza taxonomy coding

All CPG dissemination and implementation strategies were coded using the Mazza taxonomy [22]. The Mazza taxonomy was designed to assist practitioners with the classification of CPG dissemination and implementation strategies in a health care context and has been peer-reviewed and pilot-tested [23]. The Mazza taxonomy builds upon previous taxonomies, such as the EPOC Group’s data collection checklist [8], by categorizing 49 CPG dissemination and/or implementation strategies targeting HCPs into four implementation domains. First, the professional domain includes 15 strategies that target HCPs directly (e.g. *1*.*5 Educate individual health care professionals about the intent and benefit of complying with the guideline*). Second, the financial domain includes 12 strategies involving financial incentives for HCPs (*n* = 8; e.g. *2*.*1*.*1 Incentive applicable to a health care professional*) or patients (*n* = 4; e.g. *2*.*2*.*1 Incentive applicable to a patient*). Third, the organizational domain includes 18 strategies targeting organizational change at the HCP (*n* = 6; e.g. *3*.*1*.*2 Reallocated roles to assist implementation*), patient (*n* = 3; e.g. *3*.*2*.*1 Consumer feedback*, *suggestions and complaints*), or structural (*n* = 9; e.g. *3*.*3*.*1 Change in organizational structure*) levels. The fourth domain includes four regulatory strategies that involve policy and/or legislation change (e.g. *4*.*1 Change in legislation or regulation*). For reference, all Mazza taxonomy strategies are listed in Table 4.

Where classification according to a taxonomy strategy was not possible, strategies were coded as ‘Other’ under the appropriate implementation domain (e.g. *1*.*15 Other* for a strategy in the professional domain). For each study, the CPG dissemination and/or implementation strategies were coded across the experimental and control conditions to allow for the comparison of strategy effectiveness across study groups.

### Study quality

Study quality data was extracted by a single reviewer and verified by a second reviewer, with consensus being reached prior to data synthesis. The Cochrane Collaboration Risk of Bias Tool and the Cochrane Risk of Bias Assessment Tool for Non-Randomized Studies of Interventions were used to assess risk of bias in experimental and quasi-experimental study designs, respectively [24, 25].

### Data analysis

Descriptive statistics and standard deviations were used to quantify the mean number of dissemination and/or implementation strategies used in each intervention. The frequency of each implementation domain and dissemination and/or implementation strategy according to the Mazza taxonomy were also quantified [22]. Separate comparisons were made for the following: (1) single-strategy intervention vs. no intervention (i.e. no strategies used), (2) multi-strategy intervention (i.e. more than one strategy used) vs. no intervention, (3) multi-strategy intervention vs. single-strategy intervention, and (4) multi-strategy intervention vs. multi-strategy intervention. Differences in dissemination and/or implementation strategies used across experimental and comparison conditions were used to isolate distinct strategies (i.e. strategies used in the experimental condition(s) but absent from the comparison condition). These distinct dissemination and/or implementation strategies were compared to study results to determine whether they were related to positive and significant improvements in HCP behaviour and/or patient outcomes. The effectiveness of distinct strategies across differences in study outcomes (e.g. HCP only, HCP and patient, patient only), type of CPG (e.g. screening behaviour, appropriate symptom management), and the contextual factors surrounding CPG implementation (e.g. physician type, practice setting) was also examined. Due to the heterogeneity in study outcomes, a meta-analysis was not conducted.

## Results

**Fig. 1:**
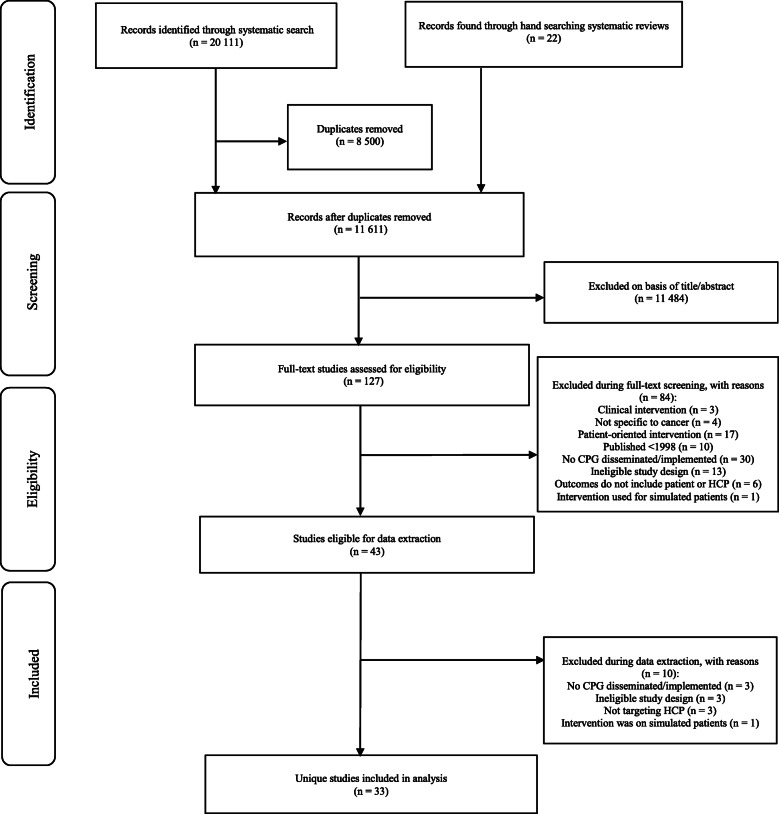
PRISMA flowchart of article selection process

The search strategy identified 20,111 records relevant to CPG dissemination and/or implementation among medical and allied HCPs in the cancer care context between 1998 and 2018. After a multi-step screening process and the removal of duplicates, a total of 34 records were eligible for inclusion. Of the 34 records included, 33 unique studies were identified, as two records described the same intervention [26, 27]. This screening process is summarized in Fig. 1.

### Participants and settings

Full details regarding study-level characteristics are reported in Table 1. Of the 33 studies, 23 were RCTs, and 10 were quasi-experimental study designs. Studies were published between 1998 and 2017. Twenty-three studies were conducted in the USA, with the remaining studies conducted in France (*n* = 3), Germany (*n* = 1), Canada (*n* = 1), the UK (*n* = 1), Australia (*n* = 1), Norway (*n* = 1), India (*n* = 1), and Italy (*n* = 1).

**Table 1:** Study characteristics, taxonomy strategies used in interventions, and summary of study outcomes

Study (country)	Study design	Participants		Setting	CPG^b^	Mazza taxonomy strategies				Outcomes^c^	
		HCP	Patients			Experimental condition^a^			Comparator condition	Primary (HCP level)	Secondary (patient-level)
Aspy et al. [28] (USA)	RCT	Primary care providers	Breast	Outpatient	Screening	1.5 Educate individual 1.12 Feedback about patients 3.1.1Additional human resources			No intervention	N/A	+ Screening rate
Ayanian et al. [29] (USA)	RCT	Primary care physicians	Colorectal	Outpatient Inpatient	Screening	1.9 Provide reminders			No intervention	N/A	+ Test completion + Detection of cancer
Bertsche et al. [30] (Germany)	QE	Physicians, nurses, pharmacists	Tumour pain	Inpatient	Appropriate symptom management	1.2 Distribute guideline 1.5 Educate individual 1.12 Feedback about patients 3.3.4 Change in technology			No intervention	Antecedent (acceptance of recommendations; N.R.) + Behaviour (deviations from guideline)	+ Symptom management
Burack et al. [31] (USA)	RCT	Primary care physicians, internal medicine, gynaecology	Cervical	Outpatient	Screening	1.9 Provide reminders Patient-directed strategy	1.9 Provide reminders		No intervention	N/A	Ø Test completion
Carney et al. [32] (USA)	RCT	Radiologists	Breast	Outpatient	Screening -	1.5 Educate individual 1.11 Feedback guideline compliance 1.12 Feedback about patients 1.15 Other—goal setting			No intervention	Ø Behaviour (recall rate)	N/A
Cohn et al. [33] (USA)	QE	Obstetrician-gynaecologists, physicians	Cervical	Outpatient	Screening	1.2 Distribute guideline 1.3 Advertise guideline 1.5 Educate individual 1.5 Educate individual 1.9 Provide reminders Patient-directed strategy	1.2 Distribute guideline 1.5 Educate individual 1.5 Educate individual 1.9 Provide reminders		No intervention	+ Antecedent (knowledge of guideline) + Antecedent (exposure to guidelines) + Antecedent (patient history)	N/A
Coleman et al. [34] (USA)	QE	Nurses, physicians, mammography technicians	Breast	Outpatient	Screening	1.5 Educate individual 1.9 Provide reminders 1.12 Feedback about patients Patient-mediated strategy			No intervention	Ø Antecedent (knowledge and attitude) Antecedent (skill; N.R.)	Ø Screening rate
Du Pen et al. [35] (USA)	RCT	Oncology nurses, oncologists	Cancer pain	Outpatient	Appropriate symptom management	1.2 Distribute guideline 1.6 Educate group 1.7 Recruit opinion leader 1.15 Other—algorithm			1.15 Other—algorithm	+ Behaviour (total provider adherence)	+ Symptom management (pain outcomes)
Du Pen et al. [36] (USA)	RCT	Oncology nurses, oncologists	Cancer pain (breast, lung, colorectal, prostate, pancreatic, ovarian, etc.)	Outpatient	Appropriate symptom management	1.2 Distribute guideline 1.15 Other—algorithm			No intervention	Behaviour (total provider adherence; N.R.)	+ Symptom management Ø Quality of life
Emery et al. [37] (UK)	RCT	General practitioners, nurses	Breast, colorectal	Outpatient	Screening	1.5 Educate individual 1.6 Educate group 1.7 Recruit opinion leader 3.3.4 Change in technology			1.2 Distribute guideline 1.6 Educate groups	Antecedent (attitude; N.R.) Antecedent (confidence; N.R.) Antecedent (barriers; N.R.) + Behaviour (screening referral)	N/A
Ferreira et al. [38] (USA)	RCT	Physicians, nurses	Colorectal	Outpatient	Screening	1.6 Educate group 1.11 Feedback guideline compliance 1.12 Feedback about patients 1.14 Feedback from health care professionals Patient-mediated strategy			No intervention	+ Behaviour (screening recommendations)	+ Screening rate
Ganz et al. [39] (USA)	RCT	Primary care providers, nurses, administrative staff	Colorectal	Outpatient	Screening	1.1 Identify barriers 1.2 Distribute guideline 1.6 Educate group 1.7 Recruit opinion leader 1.9 Provide reminders 1.14 Feedback from health care professionals Patient-directed strategy			No intervention	Antecedent (uptake of intervention; N.R.)	Ø Screening rate
Gorin et al. [40] (USA)	RCT	Primary care physicians	Breast	Outpatient	Screening	1.5 Educate individual 1.6 Educate group 1.9 Provide reminders Patient-directed strategy			No intervention	+ Antecedent (knowledge) + Antecedent (perception of barriers) + Behaviour (screening recommendation)	+ Screening rate
Hillman et al. [41] (USA)	RCT	Primary care providers	Breast, cervical, colorectal	Outpatient	Screening	1.12 Feedback about patients 2.1.2 Incentive to institution			No intervention	Ø Behaviour (screening recommendations)	N/A
Hountz et al. [42] (USA)	QE	Nurses	Colorectal	Outpatient	Screening	1.6 Educate group 1.11 Feedback guideline compliance 3.3.4 Change in technology Patient-directed strategy			No intervention	+ Behaviour (screenings ordered)	+ Test completion
Kerfoot et al. [43] (USA)	RCT	Primary care physicians, nurses, physician assistants	Prostate	Outpatient	Screening -	1.11 Feedback guideline compliance			No intervention	Antecedent (knowledge; N.R) + Behaviour (antigen screening deviations)	N/A
Lane et al. [44] (USA)	QE	Primary care physicians	Breast	Outpatient	Screening	1.5 Educate individual 1.13 Feedback from patients			No intervention	Ø Behaviour (screening referral)	N/A
Lane et al. [45] (USA)	RCT	Physicians, nurses, physician assistants	Colorectal	Outpatient	Screening	1.1 Identify barriers 1.6 Educate group 1.8 Achieve consensus 3.1.2 Reallocated roles 3.1.3 Implementation team			No intervention	+ Behaviour (screening referral)	N/A
Ling et al. [46] (USA)	RCT	Family practice, internal medicine physicians	Colorectal	Outpatient	Screening	1.1 Identify barriers 1.6 Educate group 1.10 Provide alerts 3.1.6 Other—development of protocols 3.1.6 Other—changing intervention to facilitate ease of use Patient-directed strategy			1.10 Provide alerts 1.6 Educate groups 3.1.6 Other—development of protocols Patient-directed strategy	N/A	+ Test completion
Manfredi et al. [47] (USA)	RCT	Primary care physicians	Breast, cervical, colorectal	Outpatient	Screening	1.3 Advertise guideline 1.4 Present guideline 1.6 Educate group 1.10 Provide alerts 1.11 Feedback guideline compliance 3.3.5 Change in quality assurance			1.3 Advertise guideline	N/A	+ Test completion
McDonald et al. [48] (USA)	RCT	Nurses	Cancer pain	Outpatient	Appropriate assessment/instruction practices	1.2 Distribute guideline 1.3 Advertise guideline 1.9 Provide reminders 3.1.1 Additional human resources	1.3 Advertise guideline 1.9 Provide reminders		No intervention	Ø Behaviour (pain assessment)	Ø Symptom management and quality of life
Myers et al. [26, 27] (USA)	RCT	Primary care physicians	Colorectal	Outpatient	Screening	1.1 Identify barriers 1.6 Educate group 1.9 Provide reminders 1.11 Feedback guideline compliance 1.12 Feedback about patients			1.9 Provide reminders	+ Behaviour (screening)	+ Screening rate
Ornstein et al. [49] (USA)	RCT	Primary care physicians, nurses	Colorectal	Outpatient	Screening	1.4 Present guideline 1.11 Feedback guideline compliance 1.12 Feedback about patients 1.15 Other—education on ‘best’ implementation strategies 3.1.2 Reallocated roles			1.4 Present guideline materials 3.12 Reallocated roles	+ Behaviour (method of screening)	+ Test completion
Patil et al. [50] (India)	QE	Physicians	N.R.	Outpatient	Appropriate symptom management	1.3 Advertise guideline 1.11 Feedback guideline compliance 3.3.5 Change in quality assurance 4.1 Change in legislation			No intervention	+ Behaviour (physician adherence; prescription overuse)	N/A
Phillips et al. [51] (Australia)	QE	Nurses	Lung, breast, gynaecological, colorectal, other	Inpatient	Appropriate symptom management	1.5 Educate individual 1.11 Feedback guideline compliance			No intervention	+ Antecedent (knowledge) + Antecedent (confidence) + Behaviour (pain assessment and documentation)	N/A
Raj et al. [52] (Norway)	QE	Oncologists	Breast, prostate, colorectal, lymphoma, lung, testicular, anal, upper gastrointestinal, other	Outpatient	Pain management	1.12 Feedback about patients 3.3.4 Change in technology			No intervention	Ø Behaviour (prescription)	Ø Symptom management (pain outcomes)
Rat et al. [53] (France)	RCT	General practitioners	Colorectal	Outpatient	Screening	1.12 Feedback about patients	1.11 Feedback guideline compliance		No intervention	+ Behaviour (screening)	N/A
Ray-Coquard et al. [54] (France)	QE	Physicians	Breast, colon	Outpatient	Appropriate treatment sequence (screening, procedures, continuity of care)	1.2 Distribute guideline 1.6 Educate group 1.7 Recruit opinion leader 1.8 Achieve consensus			No intervention	Behaviour (compliance with CPG; Ø breast; + colon)	N/A
Ray-Coquard et al. [55] (France)	QE	Physicians	Breast, colon	Outpatient	Appropriate treatment sequence (screening, procedures, continuity of care)	1.2 Distribute guideline 1.6 Educate group 1.8 Achieve consensus			No intervention	+ Behaviour (compliance with CPG)	N/A
Roila et al. [56] (Italy)	RCT	Oncologist	Breast, lung, ovary, colorectal, other	Inpatient	Appropriate symptom management	1.2 Distribute guideline 1.6 Educate group 1.11 Feedback guideline compliance	1.2 Distribute guideline 1.11 Feedback guideline compliance		1.2 Distribute guideline	+ Behaviour (prescription)	N/A
Sequist et al. [57] (USA)	RCT	Primary care physicians	Colorectal	Outpatient	Screening	1.9 Provide reminders Patient-mediated strategy	1.9 Provide reminders	Patient-mediated strategy	No intervention	Antecedent (attitude; N.R.)	Ø Screening rate + Detection of cancer
Walsh et al. [58] (USA)	RCT	Primary care physicians	Colorectal	Outpatient	Screening	1.1 Identify barriers 1.5 Educate individual 1.6 Educate group 1.7 Recruit opinion leader Patient-mediated strategy			1.6 Educate groups	N/A	Ø Screening rate
Wright et al. [67] (Canada)	RCT	Physicians	Colon	Outpatient	Accuracy of diagnosis	1.5 Educate individual 1.6 Educate group 1.7 Recruit opinion leader 1.9 Provide reminders			1.6 Educate groups	Ø Behaviour (staging)	N/A

*CPG* clinical practice guideline, *HCP* health care professional, *N/A* not measured in study so not applicable, *N.R.* measured in study but not reported, *QE* quasi-experimental, *RCT* randomized controlled trial, *UK* United Kingdom, *USA* United States of America

^a^Where there are two sets of strategies it indicates the presence of multiple experimental groups within a study; where strategies are repeated it indicates the coding of the same strategy in two distinct components of the intervention

^b^Direction of the recommendation is to increase behaviour, with the exception of one study as indicated with ‘–‘

^c^‘+’represents positive and significant changes and a ‘Ø’ represents a null finding

Interventions were predominantly conducted in an outpatient setting (*n* = 29), with three interventions conducted in an inpatient setting, and one conducted in both an inpatient and outpatient setting. Thirty of the 33 interventions targeted medically qualified HCPs only (e.g. primary care providers, oncologists, nurses, radiologists), and three interventions targeted both medical and allied HCPs (e.g. physician assistants, mammography technicians, pharmacists). No interventions targeted allied HCPs exclusively. Participating patient characteristics were heterogeneous; patient participants were diagnosed with, or were at risk for, various forms of cancer including colorectal (*n =* 13), breast (*n =* 9), cervical (*n =* 4), colon (*n* = 2), lung (*n* = 1), ovary (*n* = 1), prostate (*n =* 1), and unspecified (*n* = 4). Patient participants were predominately female (65%) and had a mean age of 62.16 (SD = 3.43) years.

Across the 33 included studies, 22 CPGs targeted HCP cancer screening behaviour. The remaining CPGs targeted appropriate symptom management (e.g. standards of care for pain management; *n* = 8), appropriate treatment sequence (e.g. continuity of care; *n* = 2), and accuracy of diagnosis (e.g. appropriate classification and staging of patients with cancer; *n* = 1). The majority of CPGs implemented were updates of previous guidelines (75%).

Out of the 33 studies included, 14 studies reported HCP outcomes only, 13 studies reported both HCP and patient outcomes, and six studies reported patient outcomes only. Primary outcomes (i.e. HCP behaviour) included behaviour in compliance with the CPG (*n* = 23), and/or antecedents to behaviour (*n* = 8), such as knowledge of or attitudes towards the CPG. Secondary outcomes (i.e. patient) reported included screening rate (*n* = 8), test completion (*n* = 6), symptom management (*n* = 5), detection of cancer (*n* = 2), and quality of life (*n* = 2). Although additional items regarding HCP and patient characteristics and intervention design were extracted from each study, the data presented above is reflective of items that were consistently reported across the studies included in this review.

The methodological quality of studies varied. The RCTs reviewed had moderate to high risk of bias; study quality was primarily limited by a lack of blinding and allocation concealment (see Table 2). All quasi-experimental studies were judged to have an overall serious risk of bias; study quality was primarily limited by inappropriate measurement of outcomes, missing data, and the presence of confounding variables (see Table 3).

**Table 2:** Risk of bias for randomized controlled trials

Author	Overall Risk of Bias	Confounding	Selection of Participants	Measurement of Interventions	Departures from Intended Interventions	Missing Data	Measurement of Outcomes	Selection of the Reported Result
Bertsche et al. [30]	Serious	Low	Moderate	Low	No information	Low	Serious	Moderate
Coleman et al. [34]	Serious	Low	Low	Low	No information	Serious	Serious	Moderate
Hountz et al. [42]	Serious	No information	Low	No information	No information	Low	No information	Low
Lane et al. [44]	Serious	Serious	Serious	Serious	Serious	Moderate	Moderate	Serious
Patil et al. [50]	Serious	Serious	No information	Serious	Low	Serious	Low	Low
Phillips et al. [51]	Serious	No information	Low	Low	No information	Low	Serious	Moderate
Raj et al. [52]	Serious	Low	Low	Low	No information	Low	Serious	Moderate
Ray-Coquard et al. [54]	Serious	Moderate	Low	Low	No information	Low	Serious	Moderate
Ray-Coquard et al. [55]	Serious	Moderate	Low	Low	Low	Low	Serious	Moderate

**Table 3:** ACROBAT-NRSI results for included non-randomized studies

Author	Overall risk of bias	Confounding	Selection of participants	Measurement of interventions	Departures from intended interventions	Missing data	Measurement of outcomes	Selection of the reported result
[29]	Low	Low	Low	Low	Low	Low	Low	Low
Bertsche et al. [30]	Serious	Low	Moderate	Low	No information	Low	Serious	Moderate
Coleman et al. [34]	Serious	Low	Low	Low	No information	Serious	Serious	Moderate
Hountz et al. [42]	Serious	No information	Low	No information	No information	Low	No information	Low
Patil et al. [50]	Serious	Serious	No information	Serious	Low	Serious	Low	Low
Phillips et al. [51]	Serious	No information	Low	Low	No information	Low	Serious	Moderate
Raj et al. [52]	Serious	Low	Low	Low	No information	Low	Serious	Moderate
Ray-Coquard et al. [54]	Serious	Moderate	Low	Low	No information	Low	Serious	Moderate
Ray-Coquard et al. [55]	Serious	Moderate	Low	Low	Low	Low	Serious	Moderate

### Dissemination and/or implementation strategies used

Table 4 summarizes or implementation/or implementation strategies used across all interventions. Across all 33 studies, a total of 111 strategies were used, representing 23 of the 49 Mazza taxonomy strategies [22]. The mean number of strategies per intervention was 3.25 (SD = 1.45), with a range of one to six strategies used per intervention condition (e.g. experimental or comparison). The majority (*n* = 96) of the strategies used were drawn from the professional domain, with the remaining strategies derived from the organizational (*n* = 13), financial (*n* = 1), and regulatory domains (*n* = 1). The most frequently used strategies to target HCP behaviour and/or patient outcomes included educational strategies (i.e. *1*.*5 Educate individual* (*n = 10*) and *1*.*6 Educate group* (*n* = 16)), 1.11 *Feedback guideline compliance* (*n* = 11), and *1*.*9 Providing reminders* (*n* = 10)).

**Table 4:** Frequency of Mazza taxonomy strategies used in included studies

	Random sequence generation (selection bias)	Allocation concealment (selection bias)	Blinding of participants and personnel (performance bias)	Blinding of outcome assessment (detection bias)	Incomplete outcome data (attrition bias)	Selective reporting (reporting bias)	Other bias
Aspy et al. [28]							
Ayanian et al. [29]							
Burack et al. [31]							
Carney et al. [32]							
Du Pen et al. [35]							
Du Pen et al. [36]							
Emery et al. [37]							
Ferreira et al. [38]							
Ganz et al. [39]							
Gorin et al. [40]							
Hillman et al. [41]							
Kerfoot et al. [43]							
Lane et al. [45]							
Ling et al. [46]							
Manfredi et al. [47]							
McDonald et al. [48]							
Myers et al. [27]							
Myers et al. [26]							
Ornstein et al. [49]							
Rat et al. [53]							
Roila et al. [56]							
Sequist et al. [57]							
Walsh et al. [58]							
Wright et al. [67]							

Across the 33 studies, six studies used patient-directed strategies and four studies used patient-mediated strategies as part of multi-strategy interventions targeting HCP behaviour and/or patient outcomes. More specifically, of the 14 studies that evaluated HCP outcomes only, one study used a patient-directed strategy and no studies used patient-mediated strategies. Of the 13 studies that evaluated both HCP and patient outcomes, three studies used patient-directed strategies and three studies used patient-mediated strategies. In the six studies that evaluated patient outcomes only, two studies used patient-directed strategies and one study used a patient-mediated strategy. Notably, only one study used a theory, and it was used to directly inform the design of the intervention.

### Dissemination and/or implementation strategy effectiveness

Across the body of evidence, 19 studies compared intervention(s) (2 single-strategy and 17 multi-strategy) to no intervention, five studies compared a multi-strategy intervention to a single-strategy intervention, three studies compared two multi-strategy intervention groups, and six studies compared three or four multi-strategy interventions. Due to the variability in the type of strategies across studies, and a high number of strategies used within each study, it is difficult to estimate the magnitude of impact of each individual strategy. The following provides a narrative synthesis of study results.

Positive and significant changes in HCP behaviour and/or patient outcomes were most frequently seen with two strategies: *1*.*9 Providing reminders* and *1*.*11 Feedback guideline compliance*. This held true if the strategies were used independently or if they were part of a multi-strategy intervention. For multi-strategy interventions, similar findings were found with *1*.*6 Educating group* and strategies drawn from the organizational domain (see Table 4 for list). Of note, *1*.*9 Providing reminders*, *1*.*11 Feedback guideline compliance*, and *1*.*6 Educating* group were paired with positive and significant changes in HCP and/or patient outcomes, regardless of the type of CPG (e.g. screening behaviour, appropriate symptom management), the outcome measured in the study (i.e. HCP only, HCP and patient, or patient-only outcomes) or the contextual factors surrounding CPG implementation (e.g. physician type, practice setting). The number of strategies used per intervention did not seem to influence whether positive and significant changes in HCP and/or patient outcomes were observed. Finally, interventions that incorporated patient-directed or patient-mediated strategies, or that had a basis in theory, reported no statistically significant changes in HCP behaviour and/or patient outcomes when compared to interventions without such components.

## Discussion

This review aimed to determine the effectiveness of CPG dissemination and implementation strategies among HCPs in the cancer care context. A number of notable findings about the type of HCPs targeted; the number, types, and effectiveness of CPG dissemination and implementation strategies used; and a narrative about future directions for the field will be unpacked in sequence.

High quality, safe, and patient-centred cancer care requires a multidisciplinary team of providers [15]. However, only three of the eligible studies included allied HCPs as part of their participant sample, and no studies targeted allied HCPs exclusively. Allied HCPs are valuable members of a cancer care team (e.g. providing patient education regarding self-management during treatment and follow-up [15]), and many CPGs (e.g. pain management) are applicable to a variety of HCPs, so it is surprising that they are an untapped group for research examining CPG dissemination and/or implementation. Given the increasingly important role of allied HCPs in providing patient-centred care and their presence throughout a patient’s cancer experience, it is recommended that future research examine CPG dissemination and/or implementation strategies that would be effective for this group.

Although 111 strategies were coded across the 33 studies, less than half (23/49) of the Mazza taxonomy strategies were used across all interventions. The majority of strategies used were drawn from the professional (*n* = 96) domain of the Mazza taxonomy. Consequently, there may be unused strategies for CPG dissemination and/or implementation that might be effective for improving service delivery (i.e. HCP behaviour) and/or patient outcomes in cancer care.

When considering studies that incorporated single-strategy interventions, *1*.*9 Providing reminders* and *1*.*11 Feedback guideline compliance* appear to be related to positive and significant changes in HCP behaviour and/or patient outcomes. For example, Ayanian et al. [29] used patient-specific mailed reminders (i.e. *1*.*9 Providing reminders*) to primary care physicians to improve colorectal screening behaviour. Positive and significant changes in patient outcomes of test completion and detection of cancer were observed across a 6-month period. Similarly, in Kerfoot et al. [43], physicians were asked to respond to automated clinical scenarios across a 36-week period and received immediate feedback on their clinical decision making (i.e. *1*.*11 Feedback guideline compliance*). Significant reductions in inappropriate HCP screening behaviour for prostate cancer were observed across the intervention period. Indeed, providing reminders to HCPs and feedback compliance data/information (e.g. audit and feedback strategies) are both effective for changing HCP behaviour in other clinical settings [59, 60]. For example, Cheung et al. [59] reported modest improvements in HCP behaviour following reminders in a variety of clinical settings . Similarly, Ivers et al. [60] reported small to moderate effects for the use of feedback compliance data to change HCP behaviour. Accordingly, the current review also suggests that when used independently, providing reminders to HCPs and feedback on compliance with CPGs may be effective to disseminate and implement CPGs in a cancer care setting.

The examination of distinct dissemination and/or implementation strategies across multi-strategy interventions identified *1*.*6 Educate group* as a strategy that was frequently partnered with improvements in HCP behaviour and/or patient outcomes. For example, as one aspect of their intervention, Ferreira et al. [38] delivered a 2-h group education workshop to physicians about the CPGs for colorectal screening. Positive and significant results were reported for HCP behaviour (i.e. screening recommendations) and patient outcomes (i.e. test completion). Similar to Grimshaw et al. [7], educational strategies were the most commonly used strategies in multi-strategy interventions. In the current review, educating groups was frequently paired with positive and significant changes in HCP behaviour and/or patient outcomes, in comparison to educating HCPs independently. Although passive educational interventions are common, they are unlikely to change medical or allied HCP behaviour [6, 7]. There may be additional facets to group education (i.e. incorporation of activities, discussion) which may lead to improvements in HCP and/or patient outcomes compared to educational strategies targeting individual HCPs [7]. Further, it is possible that group norms may have also contributed to the effectiveness of group educational strategies, such that HCPs were more motivated to change behaviour due to their perception of other HCP attitudes (e.g. approval/disapproval) and/or behaviours towards complying with a given CPG.

Although underutilized, strategies from the organizational domain, when implemented, were also frequently observed with positive and significant changes in HCP behaviour and/or patient outcomes. For example, Bertsche et al. [30] used an organizational strategy (i.e. *3*.*3*.*4 change in technology*) to correct physician deviation from a symptom management CPG. A computerized clinical support system was implemented to assist physicians with pain regimen recommendations for patients, with positive and significant results reported for improved pain management (i.e. patient outcomes). Current evidence suggests that organizational-level interventions in the health care context can influence clinical outcomes and efficiency [4]. Future interventions for HCPs in cancer care may benefit from multi-level approaches to HCP behaviour change by utilizing strategies from more than one implementation domain (e.g. professional, financial, organizational, and regulatory).

While not initially included in the review protocol [19], this review examined the use of patient-directed and patient-mediated strategies to support HCP behaviour change and/or influence patient outcomes. Six studies examined patient-directed strategies and four studies examined patient-mediated strategies as components in multi-strategy interventions. The findings of these studies were mixed, with little to no improvements observed for patient-directed or patient-mediated strategies when compared to interventions without such components. However, patient-directed [61] and patient-mediated [62, 63] interventions have been shown to foster HCP behaviour change and/or patient outcomes in health care settings including cancer care. The contradictory nature of the current review findings may stem from the relatively small number of studies that included patient-directed or patient-mediated strategies as intervention components. Notably, the current review’s search strategy was not designed to explicitly capture patient-directed or patient-mediated strategies; as such, other relevant studies need to be considered alongside the current findings. Consequently, additional research is needed to determine the effectiveness of CPG dissemination and/or implementation strategies that involve patients within the cancer care context.

Finally, this review identified two areas of improvement for researchers to consider to advance science and consequently practice, in the field of CPG dissemination and implementation. First, better alignment between study objectives, strategies used, and evaluation measures is required. The conceptual model of implementation research by Procter et al. [5] specifies that there are multiple levels of implementation research outcomes, including implementation outcomes (e.g. fidelity, acceptability), service outcomes (e.g. HCP behaviour), and patient outcomes (e.g. satisfaction, function). Across the 33 studies included in this review, 14 reported HCP outcomes only, 13 studies reported HCP and patient outcomes, and six studies reported patient outcomes only. Given that change must occur in proximal outcomes (e.g. attitude towards a CPG, HCP behaviour) to produce significant changes in more distal outcomes (e.g. patient function or quality of life), it is surprising that the six studies reporting patient-only outcomes did not measure or report HCP level outcomes despite explicitly targeting HCP behaviour. For example, a study by Ayanian et al. [29] provided reminders to primary care physicians to improve screening rates for persons with colorectal cancer. While the strategy targeted HCP behaviour, only a more distal patient outcome (i.e. test completion and detection of cancer) was assessed. Similarly, one study Cohn et al. [33] used a patient-directed strategy (intended to influence patient outcomes) but only reported HCP behaviour outcomes. In addition, the number of CPG dissemination and implementation strategies used per intervention did not seem to influence whether positive and significant changes in HCP and/or patient outcomes were observed, a finding that seems counter-intuitive (i.e. more strategies/intervention dose should lead to better outcomes); this finding may be accounted for by the lack of alignment between strategies used and measured outcomes. Overall, the inconsistency between study objectives, strategies used, and evaluation measures made it impossible for us to comment on which strategies are more effective for which outcomes (i.e. HCP behaviour, patient outcomes). Future work should aim to align evaluation outcomes with study objectives and strategies used, as well as measure both proximal and distal outcomes, in order to further our understanding of which CPG dissemination and implementation strategies are effective for which outcomes.

Next, given that the purpose of the studies in this review were to increase HCP behaviour in line with CPG recommendations, and strong evidence indicating that theory-based interventions lead to improved outcomes when compared to interventions that do not incorporate theory [21], the fact that only one study used theory for intervention design is surprising. In addition, 18 of the 33 included studies assessed HCP behaviour without measuring an antecedent of behaviour; seven of these studies did not find statistically significant changes in this service outcome. The paucity of theory-based interventions and evaluations in this review, as well as authors’ lack of assessments of antecedents of behaviour change, preclude conclusions regarding the interventions’ mechanism of effectiveness or ineffectiveness. Future CPG dissemination and/or implementation research should seek to incorporate theory in intervention design and evaluation so that antecedents to behaviour are considered and measured to enhance the field’s understanding of the causal mechanisms by which interventions lead, or do not lead, to HCP behaviour change and/or improved patient outcomes in the cancer care context.

### Strengths and limitations

To our knowledge, this is the first review to examine CPG dissemination and/or implementation strategies among medical and allied HCPs within the cancer care context. Several strategies were identified as potentially yielding improvements in HCP behaviour and/or patient outcomes. Further, this review highlights potential avenues for future CPG dissemination and/or implementation research (e.g. allied HCPs as the intervention targets, exploring more of the Mazza taxonomy strategies). Finally, rigorous methods were used to conduct this review, including the use of two reviewers for all data screening, extraction, and coding processes.

Some limitations should be noted. The first is the broad nature of the definition of CPG used, although similar reviews have used this definition [6, 7], it may have resulted in the inclusion of studies that disseminated and/or implemented expert opinion or recommendations (e.g. [39, 48]) which some may not see as fulfilling the criteria for a CPG. Second, to answer our research question about the effectiveness of dissemination and implementation strategies, we solely included studies that used experimental and quasi-experimental designs. By precluding cross-sectional, cohort, case, retrospective, and qualitative study designs, we likely missed examining studies that assessed antecedents to behaviour (e.g. attitudes, self-efficacy) among HCPs. Third, the heterogeneity in outcomes and the large number of strategies used across studies precluded the use of a meta-analysis, or other techniques like meta-regression, that would have allowed us to determine the unique influence of each strategy on a given outcome [64]. Further, the poor reporting of intervention details made it difficult to classify strategies according to the Mazza taxonomy, which is a frequently encountered issue when using any framework or taxonomy for data extraction (e.g. [65]). Accordingly, authors of future studies should strive to adhere to reporting guidelines (e.g. TIDieR checklist [66];) to ensure thorough descriptions of all intervention components are incorporated. Finally, the use of the Mazza taxonomy was not as straightforward as anticipated [22]. Specifically, the nuances between some strategies (e.g. the difference between an alert versus a reminder) is not explicit; thus, judgement and discussion were required by the coders. Further, many of the strategies that were described in the studies could not be classified in the Mazza taxonomy and were coded as ‘Other’. However, the Mazza taxonomy was indeed useful in deciphering promising CPG dissemination and/or implementation strategies for HCPs in the cancer care context.

## Conclusion

This review identified several CPG dissemination and/or implementation strategies that may potentially yield improvements in HCP behaviour and/or patient outcomes. Accordingly, future CPG dissemination and implementation interventions for HCPs in a cancer care context may benefit from utilizing strategies from the professional and organizational domains of the Mazza taxonomy. Future CPG dissemination and/or implementation interventions are encouraged to draw upon strategies in multiple domains of the Mazza taxonomy and explore currently unused strategies to identify additional strategies that might be effective in the cancer care context. Research in this area should aim for better alignment between study objectives, strategies used, and evaluation measures and should seek to incorporate theory in intervention design so that behavioural antecedents are considered and measured to enhance the field’s understanding of the causal mechanisms by which interventions lead, or do not lead, to changes in outcomes at all levels.

## Supplementary information

**Additional file 1.** Final search strategy for one database (Medline)

## Data Availability

All data generated or analysed during this study are included in this published article.

## Footnotes

1 This review adopted the definition of ‘allied HCP’ used in Hakkennes and Dodd [6] in order to align our findings with this previous review. Allied HCPs included the following professions: audiology, dietetics, occupational therapy, orthoptics, orthotics and prosthetics, pharmacy, physiotherapy, podiatry, psychology, radiography, speech pathology, and social work. Physicians and nurses were considered in the category ‘medical HCPs’.

2 For the purposes of this review and to remain consistent with previous studies examining CPG dissemination and implementation strategies [6, 7], an ‘intervention’ was defined as being comprised of one or more CPG dissemination and implementation strategies, delivered to a specific target audience, in a given context, at a given time.
