# Correction to: Effectiveness of guideline dissemination and implementation strategies on health care professionals’ behaviour and patient outcomes in the cancer care context: a systematic review

**DOI:** 10.1186/s13012-021-01128-w

**Published:** 2021-06-02

**Authors:** Jennifer R. Tomasone, Kaitlyn D. Kauffeldt, Rushil Chaudhary, Melissa C. Brouwers

**Affiliations:** 1 School of Kinesiology & Health Studies, Queen’s University, 28 Division Street, Kingston, Ontario Canada; 2 Department of Medicine, University of Toronto, 27 King’s College Circle, Toronto, Ontario Canada; 3 School of Epidemiology and Public Health, University of Ottawa, 600 Peter Morand Crescent, Ottawa, Ontario Canada

**Correction to: Implementation Sci 15, 41 (2020)**

**https://doi.org/10.1186/s13012-020-0971-6**

Following the publication of the original article [1], it was reported that there was an error in the order and content of Tables 2-4. The correct Tables 2, 3, 4 are given in this Correction article.

**Table 2:**
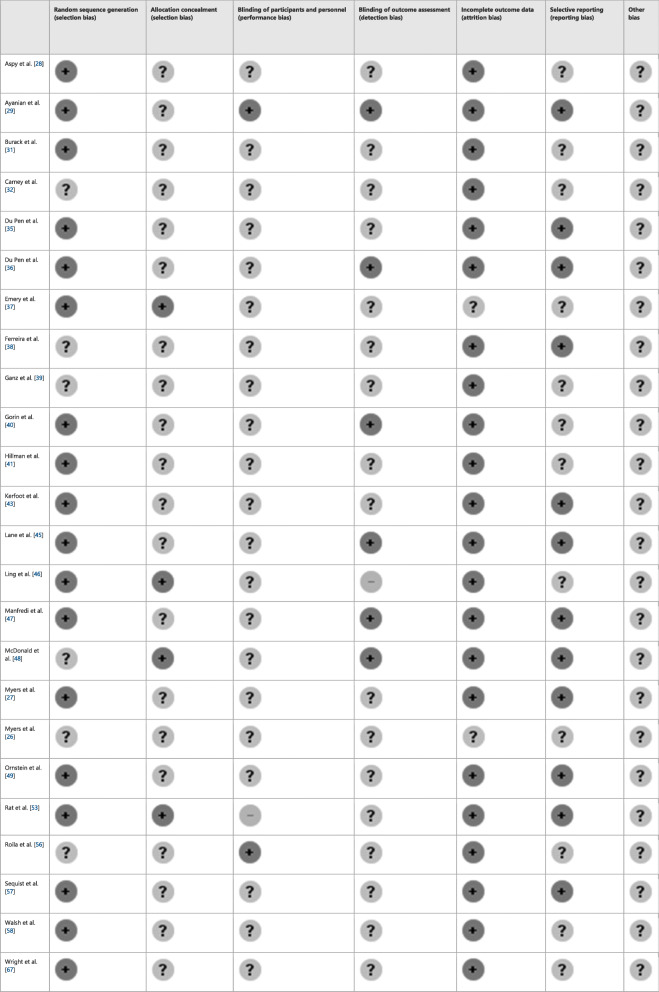
Risk of bias for randomized controlled trials

**Table 3:** ACROBAT-NRSI results for included non-randomized studies

Author	Overall Risk of Bias	Confounding	Selection of Participants	Measurement of Interventions	Departures from Intended Interventions	Missing Data	Measurement of Outcomes	Selection of the Reported Result
Bertsche et al. [30]	Serious	Low	Moderate	Low	No information	Low	Serious	Moderate
Coleman et al. [34]	Serious	Low	Low	Low	No information	Serious	Serious	Moderate
Hountz et al. [42]	Serious	No information	Low	No information	No information	Low	No information	Low
Lane et al. [44]	Serious	Serious	Serious	Serious	Serious	Moderate	Moderate	Serious
Patil et al. [50]	Serious	Serious	No information	Serious	Low	Serious	Low	Low
Phillips et al. [51]	Serious	No information	Low	Low	No information	Low	Serious	Moderate
Raj et al. [52]	Serious	Low	Low	Low	No information	Low	Serious	Moderate
Ray-Coquard et al. [54]	Serious	Moderate	Low	Low	No information	Low	Serious	Moderate
Ray-Coquard et al. [55]	Serious	Moderate	Low	Low	Low	Low	Serious	Moderate

**Table 4:** Frequency of Mazza taxonomy strategies used in included studies

Implementation Domain	Subdomains	Strategy Number and Abbreviated Strategy Name	Full Strategy Name from Mazza Taxonomy [22]	Frequency of Use
Professional		1.1 Identify barriers	Identify barriers to guideline implementation	5
		1.2 Distribute guideline	Distribute guideline materials	10
		1.3 Advertise guideline	Advertise guideline materials	4
		1.4 Present guideline	Present guideline materials at meetings	2
		1.5 Educate individual	Educate individual HCPs about the intent and benefit of complying with a guideline	10
		1.6 Educate group	Educate groups of HCPs about the intent and benefit of complying with a guideline	16
		1.7 Recruit opinion leader	Recruit an opinion leader who recommends the implementation of a guideline	6
		1.8 Achieve consensus	Achieve consensus among HCPs that the guideline is appropriate for implementation	3
		1.9 Provide reminders	Provide reminders to individual HCPs or groups about the intent and benefit of complying with a guideline	10
		1.10 Provide alerts	Provide alerts to individual HCPs or groups when clinical practice deviates from a guideline	2
		1.11 Feedback guideline compliance	Feedback guideline compliance data and information to individual HCPs or groups to improve compliance	11
		1.12 Feedback about patients	Feedback data and information about patients to individual HCPs or groups to improve compliance	10
		1.13 Feedback from patients	Feedback data and information from patients to individual HCPs or groups to improve compliance	1
		1.14 Feedback from HCPs	Feedback information from HCPs to individuals or groups to improve compliance	2
		1.15 Other		4
			**Total =**	**96**
Financial	2.1 Health care professionals	2.1.1 Incentive to HCP	Incentive applicable to a HCP	
		2.1.2 Incentive to institution	Incentive applicable available to the institution	1
		2.1.3 Grant to HCP	Grant or allowance provided to a HCP	
		2.1.4 Grant to institution	Grant or allowance provided to the institution	
		2.1.5 Penalty to HCP	Penalty applicable to a HCP	
		2.1.6 Penalty to institution	Penalty applicable to the institution	
		2.1.7 Change in reimbursement	Change in reimbursement	
		2.1.8 Other		
	2.2 Patients	2.2.1 Incentive to patient	Incentive applicable to a patient	
		2.2.2 Grant to patient	Grant or allowance provided to a patient	
		2.2.3 Penalty to patient	Penalty applicable to a patient	
		2.2.4 Other		
			**Total =**	**1**
Organizational	3.1 Health care professionals	3.1.1 Additional human resources	Additional human resources provided for implementation	2
		3.1.2 Reallocated roles	Reallocated roles to assist implementation	2
		3.1.3 Implementation team	Creation of an implementation team	1
		3.1.4 Communication between health professionals	Communication between distant health professionals	
		3.1.5 HCP satisfaction	Improved HCP satisfaction	
		3.1.6 Other		2
	3.2 Patients	3.2.1 Participation in governance	Consumer participation in governance	
		3.2.2 Consumer feedback	Consumer feedback, suggestions and complaints	
		3.2.3 Other		
	3.3 Structural	3.3.1 Change in organizational structure	Change in organizational structure	
		3.3.2 Change to setting	Change to the setting or site of service delivery	
		3.3.3 Change in physical structure	Change in the physical structure, facilities or equipment of a service	
		3.3.4 Change in technology	Change in information and communication technology	4
		3.3.5 Change in quality assurance	Change in quality assurance, quality improvement and/or performance measurement systems	2
		3.3.6 Change in delivery	Change in the method of service delivery	
		3.3.7 Integration of services	Change in the integration of services	
		3.3.8 Risk management	Change in risk management provisions	
		3.3.9 Other		
			**Total =**	**13**
Regulatory		4.1 Change in legislation	Change in legislation or regulation	1
		4.2 Change in ownership	Change in the ownership or affiliation	
		4.3 Change in licensing	Change in licensing, credentialing or accreditation of the health service and its elements	
		4.4 Other		
			**Total =**	**1**

The publisher apologizes to the authors and readers for the inconvenience.
