# Supplementary material for: Effectiveness of guideline dissemination and implementation strategies on health care professionals’ behaviour and patient outcomes in the cancer care context: a systematic review
Source: Implement Sci. 2020 Jun 3;15:41. doi: 10.1186/s13012-020-0971-6 (PMC7268663; doi:10.1186/s13012-020-0971-6)
Supplement: Supplementary file 1 — Additional file 1. Final search strategy for one database (Medline) [file 13012_2020_971_MOESM1_ESM.docx]

**Supplemental File 1: Final search strategy for one database (Medline)**

Effectiveness of guideline dissemination and implementation strategies on health care professionals’ behaviour and patient outcomes in the cancer care context: A systematic review

Tomasone, J. R., Kauffeldt, K. D., Chaudhary, R., & Brouwers, M. C.

*Implementation Science*

1 randomized controlled trials/

2 randomized controlled trial.pt.

3 controlled clinical trial.pt.

4 intervention studies/

5 experiment$.tw.

6 (time adj series).tw.

7 (pre test or pretest or (posttest or post test)).tw.

8 random allocation/

9 impact.tw.

10 intervention?.tw.

11 chang$.tw.

12 evaluation studies/

13 evaluat$.tw.

14 effect?.tw.

15 comparative studies/

16 or/1-15

17 Neoplasms/

18 Adenocarcinoma/

19 Carcinoma/

20 (adenocarcinoma* or cancer* or carcinoma* or metasta* or neoplasm* or tumo?r).ti,ab.

21 or/17-20

22 exp Practice Guidelines as Topic/

23 practice guideline?.tw.

24 (guideline? adj2 (introduc$ or issu$ or impact or effect? or disseminat$ or distribut$)).tw.

25 clinical guideline?.tw.

26 guidance.tw.

27 recommendation.tw.

28 expert opinion.tw.

29 consensus statement?.tw.

30 evidence appraisal.tw.

31 Expert Testimony/

32 or/22-31

33 academic detailing.tw.

34 ((introduc$ or impact or effect? or implement$ or computer$ or compli$) adj2 protocol?).tw.

35 ((introduc$ or impact or effect? or implement$ or computer$ or compli$) adj2 algorithm?).tw.

36 clinical pathway?.tw.

37 critical pathway?.tw.

38 Patient Education as Topic/

39 Education, Medical/

40 Clinical Audit/

41 advance directive?.tw.

42 Advance Directives/

43 Fee-for-Service Plans/

44 "Peer Review"/

45 exp education,continuing/

46 (education$ adj2 (program$ or intervention? or meeting? or session? or strateg$ or workshop? or visit?)).tw.

47 (behavio?r$ adj2 intervention?).tw.

48 pamphlets/

49 (leaflet? or booklet? or poster or posters).tw.

50 ((written or printed or oral) adj information).tw.

51 (information$ adj2 campaign).tw.

52 (education$ adj1 (method? or material?)).tw.

53 outreach.tw.

54 (opinion adj1 leader?).tw.

55 facilitator?.tw.

56 group detailing.tw.

57 consensus conference?.tw.

58 ((effect? or impact or evaluat$ or introduc$ or compar$) adj2 training program$).tw.

59 reminder systems/

60 reminder?.tw.

61 (recall adj2 system$).tw.

62 (prompter? or prompting).tw.

63 algorithm?.tw.

64 (feedback/ OR feedback.tw.) NOT ((feedback adj1 (loop? or control? or regula$ or mechanism? or inhib$ or system? or circuit? or sensory or visual or audio$ or auditory)).tw.)

65 (57 or 58) not 59

66 chart review$.tw.

67 ((effect? or impact or records or chart?) adj2 audit).tw.

68 patient education/

69 counsel$.tw.

70 compliance.tw.

71 marketing.tw.

72 exp reimbursement mechanisms/

73 fee for service.tw.

74 capitation fee/

75 "deductibles and coinsurance"/

76 cost shar$.tw.

77 (copayment? or co payment?).tw.

78 (prepay$ or prepaid or prospective payment?).tw.

79 hospital charges/

80 formular$.tw.

81 fundhold$.tw.

82 medical records/

83 medical records systems, computerized/

84 (information adj2 (management or system?)).tw.

85 peer review/

86 utilization review/

87 Mass Media/

88 Motivation/

89 incentiv$.tw.

90 allowance?.tw.

91 accreditation?.tw.

92 Licensure/

93 or/33-93

94 16 and 21 and 32 and 93

Note. Lines 1-15 contain terms relating to eligible study design; lines 17-20 contain terms relating to cancer; lines 22-31 contain terms relating to guidelines; and lines 33-93 contain terms relating to guideline dissemination and implementation interventions.
